# Significance of the Caption of a Paper or an Address

**Published:** 1897-09

**Authors:** A. W. Bitting

**Affiliations:** Purdue University, Indiana


### THE SIGNIFICANCE OF THE CAPTION OF A PAPER OR AN ADDRESS.

BY A. W. BITTING, D.V. M.,
PURDUE UNIVERSITY, INDIANA.

A CONSIDERABLE degree of importance should be attached to the selection of a proper title of a paper or the subject of an address. Veterinary literature is being searched daily by those conducting investigations along certain lines, and it is necessary for the investigator to know the complete history of the work that has preceded him. He naturally consults those works having titles pertaining to the special subject he has under consideration. Many valuable papers are passed because the title does not indicate their character, unless, perchance, he should read them or his attention be called to them by some one.

Such captions as "An Interesting Case," "An Interesting Observation," "An Experiment," "Cases from My Note-book," etc., are of frequent occurrence in our literature. There is nothing about such titles to indicate the character by which they may be classified. A perusal of the articles usually shows that a much more appropriate caption might have been selected.

Two captions by which many carefully prepared pieces of work have been buried are "A Paper," or "An Address," by Dr. ——. Not all papers or addresses are prepared upon a single topic, but the majority are, and to have them appear without an appropriate subject is to limit their usefulness to those who may read them when they are published. They do not appeal to the busy student in succeeding years. Many of the most valuable papers published in the earlier volumes of the *Veterinarian* are thus buried. The same is true, however, to a greater or less extent of all our journals, even to the present time.

Recently the writer has been preparing a card-index of the veterinary literature in his private library, and has a total of over forty thousand reference-cards. Of this number more than three thousand are useless, because of improperly selected titles. This is unfortunate, but indicates the greater care that should be exercised in the future.

Second only to a proper selection of a title is the desirability of having the full name. It is an honor to be distinguished as principal, professor, or doctor ——, and be known to the entire profession. But time passes rapidly, new members are added to the profession, and men of the same name, occupying the same or like positions become contributors to the journals, and confusion arises in ascribing some of the work to the proper author. It has already become a difficult task to prepare a bibliography of some of our well-known writers, because of using the title of his position to distinguish him, instead of his full name.

---

Iowa is said to have suffered a loss of $15,000,000 from hog-cholera alone in 1896. Surely too great a burden for any one Commonwealth to have borne in these times.

